# The system effect and group benefit equity of long-term care insurance from the perspective of short-term policy pilot

**DOI:** 10.3389/fpubh.2025.1580349

**Published:** 2025-05-30

**Authors:** Wei He, Huan Liu

**Affiliations:** ^1^School of Tourism Management, Wuhan Business University, Wu Han, China; ^2^School of Social Science, Soochow University, Suzhou, China

**Keywords:** policy pilot, long-term care insurance, disability, institutional effect, inequality

## Abstract

**Methods:**

This study focuses on the long-term care insurance (LTCI) policy pilot, using the CHARLS database to continuously track survey data. It constructs a difference in-difference model based on city, time, coverage, and beneficiaries to accurately identify policy coverage and empirically examine the institutional effect of the long-term care insurance policy pilot and the fairness of group benefits.

**Results:**

The results indicate that the policy pilot has a significant positive impact on the overall medical consumption of disabled older adults, with impacts on monthly outpatient consumption, annual hospitalization consumption, annual hospitalization times, and last hospitalization days of 0.7064, 0.4142, 0.0887, and 1.5607, respectively. In addition, the LTCI policy pilot significantly and positively affected disability-related health indicators such as individual self-assessment health, ADL disability, and the number of serious diseases, with effect sizes of 0.8677, 1.0854, and 0.6668, respectively.

**Discussion:**

The results regarding group benefit equity show that the LTCI policy pilot can improve the equity of medical consumption and disability-related health among groups; however, over time, it may exacerbate the inequality of medical consumption and disability-related health among disabled older adults in the treatment group. Based on this, the study finds that the LTCI policy pilot has effects on medical consumption and disability-related health for disabled older adults, primarily driven by the moral hazard associated with the assessed individuals obtaining LTCI treatment due to the short-term policy pilot.

## 1 Introduction

To actively address the risks associated with population aging and disability, the General Office of the Ministry of Human Resources and Social Security of the People’s Republic of China issued guidance on the pilot long-term care insurance (LTCI) system in 2016. It stated that “In the experimental stage, funds could be raised by optimizing the structure of unified account of employee medical insurance, and by transferring the surplus of employee medical insurance’s overall planning fund, as well as by adjusting premium rate of employee medical insurance. Also, it is needed to gradually establish a multi-channel financing mechanism of LTCI with mutual assistance and shared responsibility”.^1^ The national-level pilot cities for LTCI were determined at that time. On September 10, 2020, in the Guidance on Expanding the Pilot of the LTCI System issued by the National Healthcare Security Administration and the Ministry of Finance of the People’s Republic of China, 14 national-level pilot cities were designated based on the assessment of existing pilot projects.^2^ This study mainly focuses on the 15 pilot cities in the first batch for analysis. Based on the policy characteristics of the pilot areas over the past 5 years, the pilots can be divided into three categories according to the financing mechanism: The first is the “urban employee coverage mode,” the second is the “urban employee plus residents coverage model,” and the third is the “urban and rural residents coverage model.” From the perspective of the policy design of LTCI, the purpose of the pilots is to effectively improve the service security for daily life care and basic medical care for the disabled older adults, allowing them to equally access LTCI benefits if they are disabled and pass the evaluation. At the same time, because older adults are the primary consumers of medical consumption, LTCI could also help alleviate the financial pressure on the medical insurance fund. However, in practice, the LTCI policies in each pilot area have strict restrictions on coverage and beneficiaries. For example, only older adults who have received treatment related to disability for 6 consecutive months qualify, and beneficiaries must be severely disabled individuals over 60 years old. Therefore, the inclusive social insurance system often encounters institutional dilemmas. For example, although the policy design aims to be “pro-poor” or to support low-income and low-health individuals, practical obstacles often mean that the ultimate beneficiaries of the system are usually those who are high-income or in good health, showing characteristics of being “pro-rich.” Are such difficulties present in the pilot process of long-term care (LTC)? How does the experiment affect the income and health distribution among different older adults? Will differences in pilot policies lead to unequal impacts on the health of older adults? These questions are rarely discussed in existing research, yet they are critical political issues that need urgent attention. Therefore, from the perspective of policy differences in LTCI pilot areas, this study aims to deeply assess the institutional effects of China’s LTCI pilot to provide reliable evidence for further improving the policy designs of LTCI pilot areas.

The evaluation of the implementation effect of LTCI policy is a complex task. The overall path of policy evaluation involves examining the effectiveness of the pilot project from policy formulation and implementation process to implementation effect. Currently, there have been many studies on the policy formulation and implementation process of LTCI in China, but significant differences exist in the research on its implementation effect. The primary purpose of implementing LTCI is to improve the long-term care guarantee for disabled older adults and their families, thereby avoiding the dilemmas caused by LTC burdens. In existing research, such as that by Ma et al. (1) and Liu and Hu (2), preliminary studies on the implementation effect of LTCI have been conducted; however, these studies have several limitations and deficiencies. For example, their research is limited in terms of time, city, and coverage under the DID method, and does not consider the matching of beneficiaries under the LTCI pilot. This limitation hampers their ability to accurately identify the disabled individuals who truly benefited. At the same time, in terms of the health effects of LTCI, their research primarily examines the impact on the overall quality of health. From the perspective of the complexity of the LTCI system design and individual health changes, LTCI does not have a direct impact mechanism on overall health; rather, it more directly affects disability-related health indicators of severely disabled older adults. For example, the ADL disability status of disabled older adults and the diagnosis of serious diseases (e.g., most pilot areas in China have added the requirement for the number of serious diseases to the disability assessment) are key indicators. Therefore, under the existing research background, it is of great practical and policy significance to further investigate the economic benefits and health effects under the precise coverage of the pilot policy based on China’s LTCI, with the beneficiary group as the key target. In summary, this study will focus on the standards set by China’s LTCI pilot policies and their effects on the medical consumption of beneficiaries and disability-related health indicators, aiming to provide important empirical support for further optimizing the design of LTCI policies.

## 2 Literature review

As an important social policy or system, the LTCI pilot shares common characteristics with other social policies. Social policy serves as a means to address social problems, but it is also a part of these problems. In the pilot process of China’s LTCI, system coverage usually prioritizes urban residents and employees, the age limit for receiving treatment is strictly defined, and severe disability remains the main security objective. Consequently, social policies exclude rural residents, unconventional employees, and those who are non-older adults or not severely disabled (3, 4). This creates new issues of inequality. Based on current research on the effects of the LTCI system worldwide and considering the differences in policy pilots across 15 national pilot areas in China, studying the institutional effects of the LTCI policy pilot holds significant practical and theoretical importance. In addition, existing research allows us to categorize the analysis of the institutional effects of LTCI into two aspects: One is the economic or distributional effect of LTCI and the other is the health effect of LTCI.

### 2.1 Economic effect of LTCI

In terms of the economic effect of LTCI, scholars mostly focus on the effect of medical cost control (1, 5–7). The existing findings can be summarized into two parts: alternative medical expenses and released medical expenses (8). With regard to alternative medical expenses, Choi et al. (9) found that, compared with non-beneficiaries of LTCI, the number of inpatients among beneficiaries was significantly reduced, and their length of stay was significantly shortened. Ma et al. (1) and others found that LTCI effectively saved expenditures from the medical insurance fund. In terms of released medical expenses, research shows that in developed countries, regardless of the model of LTC policy adopted, the total cost of LTC social insurance expenditure is rising. For example, Schut and Berg’s (10) estimation demonstrates that the proportion of LTCI expenses in GDP in the Netherlands is increasing. This result is also supported by Swift et al. (11), Theobald and Hampe (12), and Shimizutani (13), who found that the financial burden rate of total costs has gradually increased in Germany and Japan. Liu (14) and Liu and Hu (2) also identified this trend in China. In terms of personal burden, in most countries that implement LTCI, beneficiaries need to pay a certain proportion of the costs (15–17). For example, the research of Rothgang (18), Rapp et al. (19), and Nadash and Shih (20) shows that a high proportion of self-pay or a high proportion of LTC expenses significantly impacts the personal burden of beneficiaries and increases medical consumption behavior. The research of Boo et al. (5) on health care utilization and nursing costs for LTC beneficiaries in their final year of life shows that in Korea, a considerable number of LTCI beneficiaries die within one year of receiving benefits. Individuals who use both medical care services and LTC services incur the highest medical costs in their final year of life, and this cost increases as death approaches, with about half of the participants dying in hospitals. In addition, the utilization rate of LTC services has increased from 13.0 to 22.8% until older adults die, while the rate decreased from 34 to 20% if older adults die at home. In summary, the utilization of LTC services did not reduce medical costs by replacing unnecessary hospitalizations. Furthermore, research on hospice care for older adults in China by Zhang et al. (21) found that the total costs and direct costs paid by the government or insurance fund were significantly higher than those paid by the original family.

### 2.2 Health effects of LTCI

There are few studies on the health effects of LTCI, and a consistent conclusion has not been reached. The related studies can be summarized into two parts. The first part focuses on the positive health effects of LTCI. For example, Yasutake et al. (22) estimated the effectiveness of the LTCI intervention policy from the perspective of a community-based oral health and nutrition plan (OHN plan) in Japan’s LTCI system. Their results show that the OHN plan is effective and has achieved the goal of reducing accidental disability and medical expenses. Oriented by the concept of “value medical treatment,” Ma et al. (1) found that LTCI did not save medical expenses but improved mental health and reduced physical pain to a certain extent. However, their study has major defects, as it only took Qingdao, China, as a sample for analysis. The second part concerns the negative health effects of LTCI. For example, several scholars point out that medical security or LTC service policy is not as important as expected (23, 24). The health effects of medical security are actually very weak compared to other factors such as genes, environment, region, and income (25). A few scholars also analyzed the effects from the perspective of health behavior. They studied whether older adults can improve their health level through the utilization of LTC services after the implementation of the LTCI system. For example, Jung and Yim (26) found that from 2007 to 2009, total medical expenses, medical expenses of geriatric hospitals, length of stay, and annual length of stay in geriatric hospitals are significant factors affecting the utilization of LTC, which restricts the effect of LTCI on the health improvement of older adults. Boo et al. (5) also confirmed this conclusion and demonstrated that older adults have a higher utilization rate of LTC services near their death. Nemoto et al. (27) focused on the differences in physical vulnerability of people covered by LTCI. They found that the physical function of vulnerable older adult individuals was worse than before the onset of disability.

From the literature review, it is evident that existing research on the effect of the LTCI system is primarily macro-level analysis, mainly focusing on LTCI in developed countries. Although a few scholars have also analyzed the effect of medical cost control in China from a micro viewpoint, the samples have been limited, as seen in the study by Ma et al. (1). Thus, there are deficiencies in the applicability of the existing research conclusions. At the same time, due to limitations in research methods and data, as well as the insufficient matching of actual beneficiaries of long-term care insurance policies in China’s pilot areas, the research conclusions have significant limitations in applicability. In addition, existing research mainly focuses on its role in medical cost control, and evaluations of the overall institutional effect are absent (28–30). In this regard, referencing the research of Liu and Hu (31) and Liu and Wang (32), this study analyzes the first batch of 15 national LTCI pilot areas in China. Using the DID method, it precisely matches the beneficiaries affected by the policy and uses three phases of follow-up survey data from the China Health and Retirement Longitudinal Study (CHARLS). This allows for a deep investigation of the institutional effect of the LTCI pilot, providing reliable support for the adjustment and optimization of LTCI pilot policies. The main contributions of this study are as follows: (1) By taking the three types of policy pilots that emerged during the experiments in the 15 national LTCI pilot cities as the focus, this study defines them as a sample of policy implementation and accurately constructs a treatment group sample based on city, time, coverage, and beneficiaries. In conjunction with the characteristics of specific pilot policies, it examines the impact of the policy pilot on the medical consumption of beneficiary groups, disability-related health indicators, and its impact on the fairness of group system benefits, thereby expanding the existing research perspective and enriching the research content of long-term care insurance policies. (2) In terms of research methods, we continue to use the DID method for sample matching. However, based on the existing scholars’ oversight of the precise matching of policy beneficiary groups, we attempt to build a treatment group using core indicators such as city, time, coverage, and beneficiaries to precisely match the actual beneficiaries under the pilot LTCI policy, thereby addressing the limitations of existing research regarding the policy effects on beneficiaries. In addition, we also use the concentration index and the Theil Index to investigate the fairness of group benefits under different overall planning models, providing important empirical support and insights for optimizing the overall planning model of policies.

## 3 Methods

### 3.1 Benchmark model design

This study uses a panel model to identify the impact of differences in LTCI pilot policies on the equity of medical consumption and health levels of older adults. The benchmark model is set as follows:


(1) $$Y_{\mathrm{ijt}}=\alpha +\alpha_{1}\text{Trea}t_{\mathrm{ijt}}+\beta X_{\mathrm{ijt}}+\omega_{t}+\delta_{i}+\varepsilon_{\mathrm{ijt}}$$

In Equation 1, *_i_* represents the individual, *_j_* indicates the group, *_t_* represents time, and $Y_{\mathrm{ijt}}$ represents the explained variable, which is the outcome variable of grouping. In this study, the variable includes both medical consumption and disability-related health indicators, which represent the economic effect and health effect, respectively. Theoretically, in addition to the indirect effect on controlling basic medical expenses, the direct economic effect of LTCI is mainly reflected in the release of the labor force from disabled families, the improvement of employment in regional LTC services, the promotion of the pension service industry, etc. Thus, LTCI plays a positive role in regional economic growth. However, considering the short implementation time of LTCI in the 15 national pilot cities and the frequent dynamic adjustments in the standards for designating LTC service institutions, its direct economic effect is not obvious. Based on this, and referring to existing methods for evaluating the effects of LTCI policies, we attempt to establish an economic effect model centered on medical consumption. Here, we select monthly outpatient consumption, monthly outpatient times, annual hospitalization consumption, annual hospitalization times, and last hospitalization days as key indicators. Theoretically, the health effect of LTCI is mainly reflected in its positive effect on changes in the disability status of disabled individuals. Through policy intervention, it is possible to improve the disability status of individuals or maintain the current level of disability, thereby minimizing the economic risks associated with worsening disability. Rehabilitation nursing plays a crucial role. From a practical perspective, most individuals covered by the current policy pilot are severely disabled, and the LTC service projects primarily focus on basic daily life care. Therefore, combining theory and practice, the pilot policy of LTCI has certain health effects, with the health effects associated with disability being the most significant. Based on this, we refer to Grossman's (33) health utility model to construct the health effect model of LTCI. We tested individual self-assessment health status, the number of serious diseases, and ADL disability levels as important health indicators related to disability to examine health impacts from different dimensions. Among them, self-assessment health is a comprehensive reflection of an individual’s health level; the number of serious diseases is an important reference indicator for assessing the level of disability in most pilot areas; and the ADL disability level reflects changes in individuals’ ability to care for themselves in daily life due to institutional differences.

In Equation 1, $\text{Trea}t_{\mathrm{ijt}}$ represents the processing sample, which is obtained according to the city, time, coverage, beneficiaries and other indicators, that is, $\text{Trea}t_{\mathrm{ijt}}$ = (City×Time). As the pilot cities of LTCI in China are not fully covered by the system, and the beneficiaries are mainly the severely disabled, that is, City = (coverage×Beneficiaries). The coefficient $\alpha_{1}$ is the focus of this study. Here, City refers to the pilot city for LTCI and Time refers to the year when the LTCI pilot was carried out.

Coverage refers to the specific groups covered by LTCI in pilot areas. By sorting out the policies of 15 national LTCI pilots in China, the pilot LTCI policies can be divided into three categories: The first category only covers insured urban employees by basic medical insurance, such as Chengde, Qiqihar, and Ningbo; the second category covers urban employees and urban residents who participate in medical insurance, such as Changchun; and the third category includes not only the urban population but also the rural population, such as Shanghai and Suzhou. According to the availability of data and the coverage of the CHARLS survey, we compile the data of the three types of pilot cities. Because the CHARLS survey area does not include Changchun, Nantong, and Shihezi, the policy pilots are further divided into three types here: The first is the city that did not implement the policy, defined as 0, indicating non-policy coverage. The second type consists of cities where the pilot only covers urban workers, defined as 1, while others are defined as 0. The third type includes cities where pilots have full coverage of urban and rural residents, with the corresponding residents defined as 1, indicating they are covered by the policy. Qingdao is a special case; it initiated the pilot of LTCI in 2011, but the system only covered rural residents until 2015. Therefore, the policy pilot categories are defined as treatment groups according to these time points. Referring to the collection and payment methods of LTCI in pilot cities, we determine individual’s participation in LTCI in different areas based on the types of basic medical insurance they have. Urban employees are identified based on their participation in employee medical insurance, while urban and rural residents are categorized according to the basic medical insurance for urban residents, the new rural cooperative medical system, and the medical insurance for urban and rural residents.

Beneficiaries refer to the disabled individuals who are entitled to treatment mainly guaranteed by the policies in the pilot city, and the policy beneficiaries are shown in Table 1. Because CHARLS does not have a direct indicator of the degree of disability, and the disability assessment standards in the pilot city are not completely consistent, we chose to refer to the Barthel simple scale to assess the disability level of the disabled individuals. At the same time, the pass rate of the simple disability assessment scale in each pilot city is about 70% ~ 80%, so the simple scale used in this study can accurately reflect the real situation in the pilot area. Finally, according to the results of the ADL disability assessment, the level of severe disability is defined as 1, while in Guangzhou, Suzhou, Qingdao, and Qinghai, the corresponding degrees of partial severe disability or moderate disability are also defined as 1 based on the situation of the beneficiaries, indicating that they are beneficiaries, while others are defined as 0, indicating that they are non-beneficiaries.

**Table 1:** LTCI policy characteristics of 12 pilot cities in China.

Type	Co-ordinating type	Financing mechanism	Evaluation criteria	Treatment guarantee	Target group
Chengde	Employee coverage	Constant ratio mode	Refer to Barthel scale	Payment by service type	Severe disability
Qiqihaer	Employee coverage	Quota mode	Refer to international assessment scale	Payment by service type	Severe disability
Ningbo	Employee coverage	Others	Refer to international assessment scale	Payment by service type	Severe disability
Anqing	Employee coverage	Quota mode	Refer to Barthel scale	Payment by service type	Severe disability
Guangzhou	Employee coverage	Quota mode	Refer to international assessment scale	Payment by service type	Needs assessment reaches levels 1–3
Chongqing	Employee coverage	Quota mode	Refer to Barthel scale	Payment by agreement	Severe disability
Chengdu	Employee coverage	Constant ratio mode	Localization evaluation criteria	Payment by service type	Severe disability
Suzhou	Employee and urban–rural coverage	Quota mode	Localization evaluation criteria	Payment by service type	Moderate or severe
Shangrao	Employee and urban–rural coverage	Quota mode	Localization evaluation criteria	Payment by service type	Severe disability
Qingdao	Employee and urban–rural coverage	Constant ratio mode	Localization evaluation criteria	Payment by insured population	Needs assessment is levels 3–5
Jingmen	Employee and urban–rural coverage	Constant ratio mode	Refer to international assessment scale	Payment by service type	Severe disability
Shanghai	Employee and urban–rural coverage	Constant ratio mode	Localization evaluation criteria	Payment by service type	Needs assessment reaches levels 2–6

The policies in this table come from the policy documents of each pilot city (sorted by the author). In the financing mechanism, “quota mode” refers to that each person paying fees according to a fixed standard every year, while “constant ratio mode” refers to the allocation from the personal medical insurance fund in proportion. Others refer to the allocation of a part of the fund as the initial LTCI start-up fund. “Payment by service type” in the treatment guarantee refers to the corresponding cost compensation according to institutional care and home care; “Payment by insured population” refers to the differentiated LTCI reimbursement rate determined according to the identity of employees or urban and rural residents; “Payment by agreement type” refers to the payment of nursing expenses according to fixed-point agreement institutions and non-fixed-point agreement institutions.

Finally, according to the interaction of city, time, coverage, and beneficiaries, the samples were processed to obtain the groups actually affected by the pilot policy of LTCI. The statistical results are shown in Table 2. About 1.13% of the samples were affected by the policy, meaning that 282 samples were influenced by the policy.

**Table 2:** Descriptive statistics of variables.

Variable	Definition	Sample	Mean	SD	Min	Max
Treat	Control group = 0, treatment group = 1, that is, the beneficiaries of the policy protection in the policy coverage area	25,063	0.0113	0.1055	0	1
Registered residence	Urban = 1, rural = 0	25,063	0.3889	0.4875	0	1
Medical insurance	If an individual participates in any medical insurance, it will be recorded as 1, and if not, it will be recorded as 0	24,814	0.9885	0.1066	0	1
Age	Select people aged 60 and above, that is, the actual age of the survey year	25,063	68.0666	6.6480	60	115
Gender	Male = 1, female = 0	25,063	0.4938	0.5000	0	1
Death of spouse	Death of spouse = 1, other = 0	25,063	0.1977	0.3983	0	1
Education level	1 ~ 10, respectively, indicate that the level of education is getting higher and higher, based on actual diploma	25,063	3.1005	1.3513	1	10
Pain perception	1 ~ 5, respectively, indicate more and more pain	25,063	1.9501	0.9119	1	5
Degree of depression	1 ~ 4, respectively, indicate that the degree (time) of depression is increasing	25,063	2.3588	0.8100	1	4
Family income level	1 ~ 5, respectively, indicate that the family income level is getting lower and lower	23,904	2.7034	0.7837	1	5

The specific values of education level are set as follows: 1 = no formal education (illiterate); 2 = Did not finish primary school; 3 = elementary school (include Sishu/home school); 4 = middle school; 5 = high school; 6 = vocational school; 7 = 2-/3-year college/associate degree; 8 = 4-year college/bachelor’s degree; 9 = master’s degree; 10 = doctoral degree/Ph.D.

In addition, in Equation 1, $X_{\mathrm{ijt}}$ represents individual covariates, which include demographic characteristic variables such as gender, age, and marital status; as well as socioeconomic status variables such as education level and family income level, along with self-assessment health, ADL disability, the number of serious diseases, and other disability-related health variables. $\omega_{t}$ and $\delta_{i}$ in the benchmark model represent time and individual fixed effects, respectively, and $\varepsilon_{\mathrm{ijt}}$ represents the random disturbance term.

### 3.2 Measurement indicators of inequality

The concentration index and Theil Index are selected as the key indices of inequality to investigate the fairness of medical consumption and disability-related health levels with different income levels under different LTCI policies.

#### 3.2.1 Concentration index

The concentration index is consistent with the Gini coefficient, which generalizes the Lorenz curve. It reflects the variation in the proportion of resources occupied by people of different income levels, and its value range is −1 ~ 1. In the trend chart, the concentration index appears as a curve from left to right, reflecting the distribution of occupied resources among different proportions. In the case of absolute equality, its slope is 45. In general, the calculation formula for the concentration index, which reflects the area below the slash, is


(2) $$S=(1/2)\sum_{j=1}^{j}(B_{j-1}+B_{j})(A_{j-1}+A_{j})$$

In Equation 2, where $A_{j}$ is the cumulative percentage of population of group *j* and $B_{j}$ is the cumulative percentage of statistical indicators of group *j*, such as the cumulative percentage of medical consumption and health level in this study. The meaning of the index indicates that if the concentration index is positive, it suggests that medical consumption and a high health level benefit high-income groups, resulting in a reverse (regressive) distribution effect. The larger the coefficient, the more “pro-rich” the institutional effect becomes. Conversely, if the concentration index is negative, it implies that medical consumption and a high health level benefit low-income groups, indicating a positive (progressive) distribution effect; the smaller the negative value, the more “pro-poor” the institutional effect.

#### 3.2.2 Theil Index

The Theil Index is well-known for measuring income inequality between individuals or regions. It is also referred to as Theil’s entropy measure. In terms of computational advantage, the Theil Index can not only reflect group inequality but also measure the contributions of within-group gaps and between-group gaps to the total gap. Unlike the Gini coefficient, which is highly sensitive to the inequality among middle-income individuals, the Theil entropy T index, L index, and V index are more responsive to changes in upper- and lower-income groups, respectively. Thus, they are complementary. Here, they are defined as follows:


(3) $$\begin{array}{l} T=\frac{1}{n}\sum_{i=1}^{n}\frac{y_{i}}{\bar{y}}\log(\frac{y_{i}}{\bar{y}})=T_{b}+T_{w}=\\ \sum_{k=1}^{k}y_{k}\log\frac{y_{k}}{n_{k}/n}+\sum_{k=1}^{k}\\ y_{k}(\sum_{i\in g_{k}}\frac{y_{i}}{y_{k}}\log\frac{y_{i}/y_{k}}{1/n_{k}}) \end{array}$$

where $y_{i}$ represents the income or health level of individual *_i_* and $\bar{y}$ represents the average income or health level of all the individuals. $T_{b}$ and $T_{w}$ are the decomposition of the Theil Index, representing inequality between groups and within groups, respectively. The rightmost side of Equation 3 calculates the decomposition term of the Theil Index. Generally speaking, a smaller Theil Index indicates a lower degree of inequality, and vice versa.

### 3.3 Sample selection criteria

The LTCI policy pilot characteristics of the first batch of 12 pilot cities in China are shown in Table 1. The CHARLS survey data covers 12 of China’s first batch of LTCI pilot cities in 2016 (a total of 15), with only Changchun, Nantong, and Shihezi not included. Therefore, only 12 pilot cities are analyzed in this study. The treatment group was determined according to the overall planning category in the second column, the evaluation criteria in the fourth column, and the beneficiaries in the last column of Table 1. It can be seen from the 3rd to 5th columns in Table 1 that the pilot cities with “employee coverage” as the main focus also show potential consistency in terms of financing mechanism, evaluation criteria, and treatment guarantees, while the pilot cities with “employee & urban–rural coverage” exhibit similar features. Therefore, it is feasible to define different pilot categories as LTCI.

## 4 Data

### 4.1 Data collection

The data for this study are obtained from the CHARLS survey data from 2013, 2015, and 2018. The CHARLS data cover samples from 28 provinces, municipalities, and autonomous regions in mainland China. The survey targets the population aged 45 and over, which reflects the basic demographic characteristics of China’s older adults. This study selected three periods of follow-up survey data for analysis. After data screening and selection, we focus on individuals aged 60 and above, with those having mild disabilities as the primary subjects, resulting in 25,063 valid samples and 23,904 statistical samples of family income. In the process of revising, we chose the mean substitution and interpolation method to compare and analyze the subjects with the survey samples from adjacent areas, and then filled in the gaps. The definitions and statistics of the core explanatory variable (LTCI pilot model) and related individual covariates are shown in Table 2.

### 4.2 Main variables and descriptive statistics

In addition, ADL in this study is obtained from the six questions DB10-DB15 in the “Functional Limitations and Helpers” section of the CHARLS questionnaire. The corresponding questions are “Do you have difficulties in dressing, bathing, eating, getting in and out of bed, going to the toilet, and controlling your urine and urine?” The corresponding options are 1–4: “1 = No, I do not have any difficulty; 2 = I have difficulty but still can do it; 3 = Yes, I have difficulty and need help; 4 = I cannot do it,” which are regarded as the main indicators of ADL. In addition, since the total score of ADL ranges from 6 to 24, a higher total score indicates a more severe degree of disability. For the convenience of analysis, we divide the ADL score into five levels, representing different disability levels: 6 points is level 1, indicating health; 7–9 points is level 2, indicating mild disability; 10–14 points is level 3, indicating moderate disability; 15–20 points is level 4, indicating partial severe disability; and 21–24 points is level 5, indicating severe disability (4).

Tables 3, 4 provide the descriptive statistics of the core explanatory variables in this study: medical consumption and disability-related health level. Table 3 shows that in the control group, older adults have significant urban–rural differences in monthly outpatient consumption, annual hospitalization consumption, and annual hospitalization times. For example, the monthly outpatient consumption of urban older adults is 801.004 yuan per person per month higher than that of rural older adults. In the treatment group, the difference in related medical consumption between urban and rural residents was significantly reduced. In general, compared with urban residents, the medical consumption of rural residents has also increased significantly, and is higher than it was before the pilot project.

**Table 3:** Descriptive statistics of medical consumption groups.

Variable	Sample: before treatment				
	Rural (15255)		Urban (9526)		Mean difference
	Mean	SD	Mean	SD	
Monthly outpatient consumption (yuan)	1490.577	8410.399	2291.581	9979.670	−801.004***
Monthly outpatient times (times)	0.478	1.543	0.440	1.564	0.038*
Annual inpatient consumption (yuan/year)	856.160	7350.495	1438.327	12349.240	−582.167***
Annual inpatient times (times)	0.280	0.854	0.315	0.850	−0.035***
Last hospitalization days	1.997	7.710	2.453	7.747	−0.456***

Variable	Sample: after treatment				
	Rural (62)		Urban (220)		Mean difference
	Mean	SD	Mean	SD	
Monthly outpatient consumption (yuan)	10696.94	29439.41	10362.11	39834.200	334.826
Monthly outpatient times (times)	1.226	4.313	0.645	2.516	0.580
Annual inpatient consumption (yuan/year)	3112.903	10458.12	13493.94	45776.150	−10381.037*
Annual inpatient times (times)	0.613	0.930	0.900	1.496	−0.287
Last hospitalization days	8.823	24.402	5.873	9.259	2.950

Number of samples is given within brackets, ^***^p < 0.01, ^*^p < 0.1.

**Table 4:** Descriptive statistics of disability-related health-level grouping.

Variable	Sample: before treatment				
	Rural (15255)		Urban (9526)		Mean difference
	Mean	SD	Mean	SD	
Self-assessment health	3.0954	0.8069	2.9688	0.7759	0.1266***
ADL disability	2.1117	0.6760	2.1852	0.5828	−0.0735***
Number of serious diseases	0.2682	0.7268	0.3271	0.8427	−0.0589***

Variable	Sample: after treatment				
	Rural (62)		Urban (220)		Mean difference
	Mean	SD	Mean	SD	
Self-assessment health	3.4945	0.9220	3.5344	0.8337	−0.0399
ADL disability	2.6613	0.8287	2.5000	0.8137	0.1613
Number of serious diseases	1.0645	1.2915	1.2273	1.3492	−0.1628

Number of samples is given within brackets, ^***^p < 0.01, ^**^p < 0.05, ^*^p < 0.1. The value of self-rated health is 1 ~ 5, which means that it decreases from very good to very bad, respectively. The number of serious diseases refers to the total number of major diseases diagnosed in older adults, including malignant tumors, heart disease, and stroke. The larger the value, the worse the health quality.

Table 4 of descriptive statistics for disability-related health level groups shows a significant difference in health levels between urban and rural older adults in the control group. Specifically, the self-assessment health level of rural older adults is worse than that of their urban counterparts, while the degree of ADL disability and the number of serious diseases are higher than urban older adults. In the treatment group, there is no significant difference in disability-related health levels between urban and rural older adults. The average values for self-assessment health and serious diseases are higher for urban older adults, but the degree of ADL disability is lower than rural older adults. The following section will further discuss the equity of group benefits of the LTCI policy pilot based on the concentration index and the Theil Index.

### 4.3 Test of the parallel trend assumption

**Figure 1:**
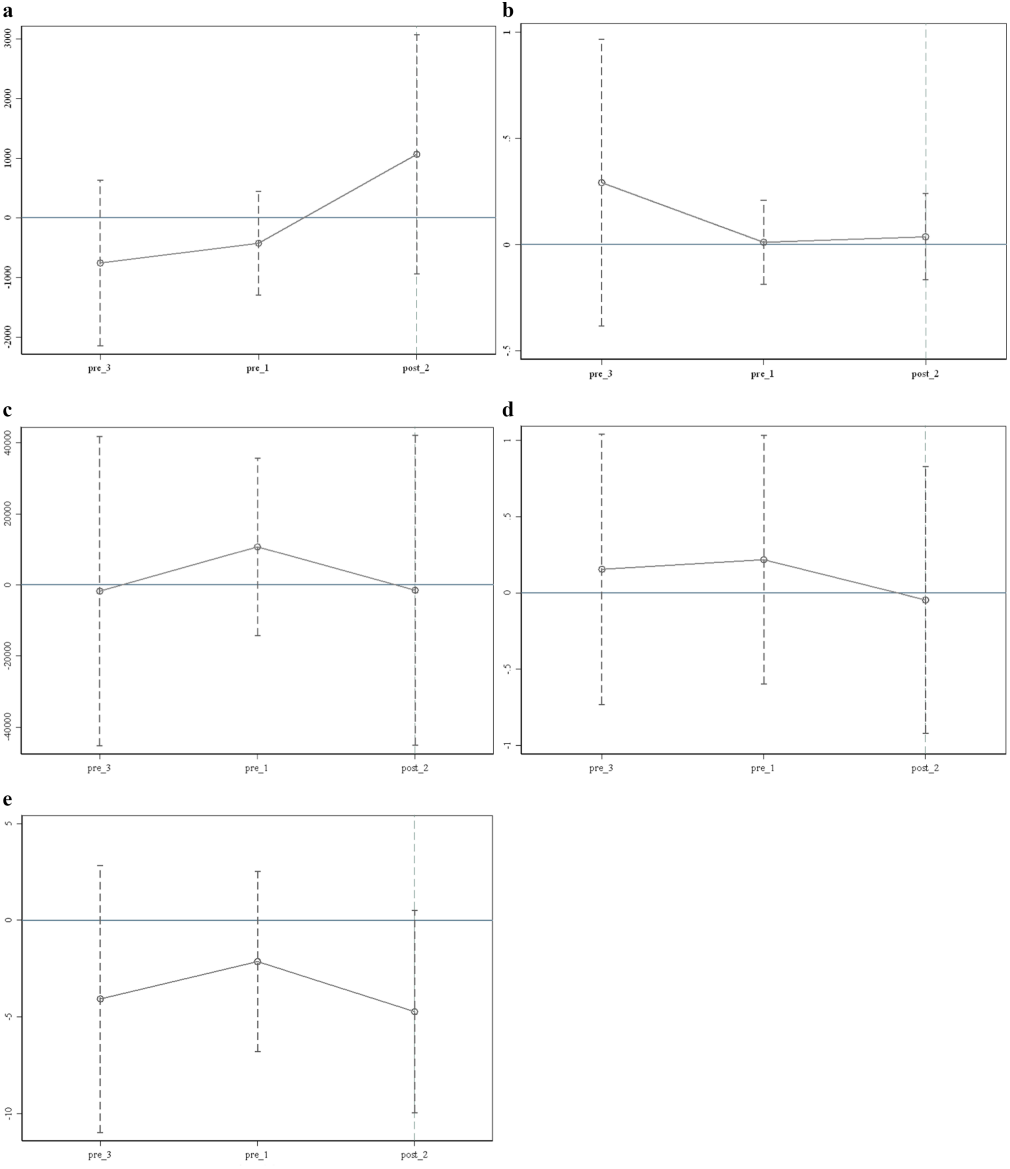
Test of the parallel trend assumption. **(a)** Test 1 of the parallel trend assumption (Monthly outpatient consumption). **(b)** Test 2 of the parallel trend assumption (Monthly outpatient times). **(c)** Test 3 of the parallel trend assumption (Annual inpatient consumption). **(d)** Test 4 of the parallel trend assumption (Annual inpatient times). **(e)** Test 5 of the parallel trend assumption (Last hospitalization days). Each vertical axis in the figure represents the trend of core variables affected by the policy change. The horizontal axis represents the time points before and after the implementation of the policy, with pre_3 and pre_1 indicating the third and first phases before the policy’s implementation, corresponding to 2013 and 2015, respectively; post_2 refers to the second period after the policy’s implementation, corresponding to 2018 in this article. The data in this work do not include 2014, 2017, and 2019, so the corresponding pre_2, post_1, and post_3 do not exist.

To ensure that the benchmark model meets the basic requirements for the use of the DID model and accurately reflects the effect of the LTCI policy, we first test the parallel trend of the core variables in this paper before the benchmark analysis. The results are shown in Figure 1. Figures 1a–e illustrate monthly outpatient consumption, monthly outpatient times, annual inpatient consumption, annual inpatient times, last hospitalization days, and other core variables.

It can be seen from Figure 1 that the trend of relevant variables reflecting the medical consumption of disabled individuals before the policy’s implementation is significantly different from that after it. For example, the trend from pre_3 to pre_1 differs significantly from that of pre_1 to post_2. Therefore, the parallel trend test results indicate significant differences in the medical consumption of beneficiaries before and after the policy’s implementation, confirming that the core data in this paper meet the common trend test. In addition, the test results for key health levels are consistent with these findings.

## 5 Results

### 5.1 Economic effects of LTCI policy pilots

#### 5.1.1 Medical consumption effect

##### 5.1.1.1 Benchmark model test

Table 5 presents the benchmark model test results, which indicate that the pilot LTCI policy has significant positive effects on monthly outpatient consumption, annual hospitalization consumption, annual hospitalization times, and last hospitalization days, with impact effects of 0.7064, 0.4142, 0.0887, and 1.5607, respectively. However, its impact on monthly outpatient times is not significant. This result shows that, compared with the control group, the pilot LTCI policy in the treatment group has significantly increased monthly outpatient consumption by 70.64%, annual hospitalization consumption by 41.42%, annual hospitalization times by 0.0887, and last hospitalization days by 1.5607 days.

**Table 5:** Results of benchmark model test and city fixed-effects test.

Variable	Explained variable: medical consumption				
	Monthly outpatient consumption (1)	Monthly outpatient times (2)	Annual inpatient consumption (3)	Annual inpatient times (4)	Last inpatient days (5)
Treat	0.7064*** (0.2164)	0.1246 (0.1032)	0.4142*** (0.1364)	0.0887* (0.0519)	1.5607*** (0.4721)
Control variable	Control	Control	Control	Control	Control
R-square	0.5611	0.5172	0.5364	0.4554	0.4637
Observations	23,690	23,690	23,690	23,690	23,690
Sample of groups	10,625	10,625	10,625	10,625	10,625

Variable	City fixed effect				
Treat	0.7313*** (0.2184)	0.1576 (0.1040)	0.4319*** (0.1377)	0.0961* (0.0523)	1.6904*** (0.4774)
City fixed effect	Control	Control	Control	Control	Control
Control variable	Control	Control	Control	Control	Control
R-square	0.5745	0.5332	0.4485	0.4675	0.4472
Observations	23,690	23,690	23,690	23,690	23,690

Number of samples is given within brackets, ***p < 0.01, *p < 0.1. During data processing, we applied logarithm transformations to monthly outpatient consumption and annual hospitalization consumption. The control variables in the model mainly include the individual health variables such as ADL disability, number of serious diseases, self-assessment health, degree of depression, physical pain, as well as the covariates reflecting individual characteristics such as age, death of spouse, education level, and gender, as noted below.

As there are many differences in the comprehensive characteristics of each pilot city during the pilot process, we fixed the city impact effect to reduce the error in the estimation results caused by these differences based on the benchmark model test. The lower part of Table 5 presents the results of the city fixed-effects test, which shows that treatment still has a significant impact on monthly outpatient consumption, annual hospitalization consumption, annual hospitalization times, and last hospitalization days, indicating that city characteristics do not affect the benchmark test results.

##### 5.1.1.2 Robustness test

To ensure the reliability of the benchmark test results, we conducted a series of robustness tests in further research, including a replacement test and a screening control group test.

**Figure 2:**
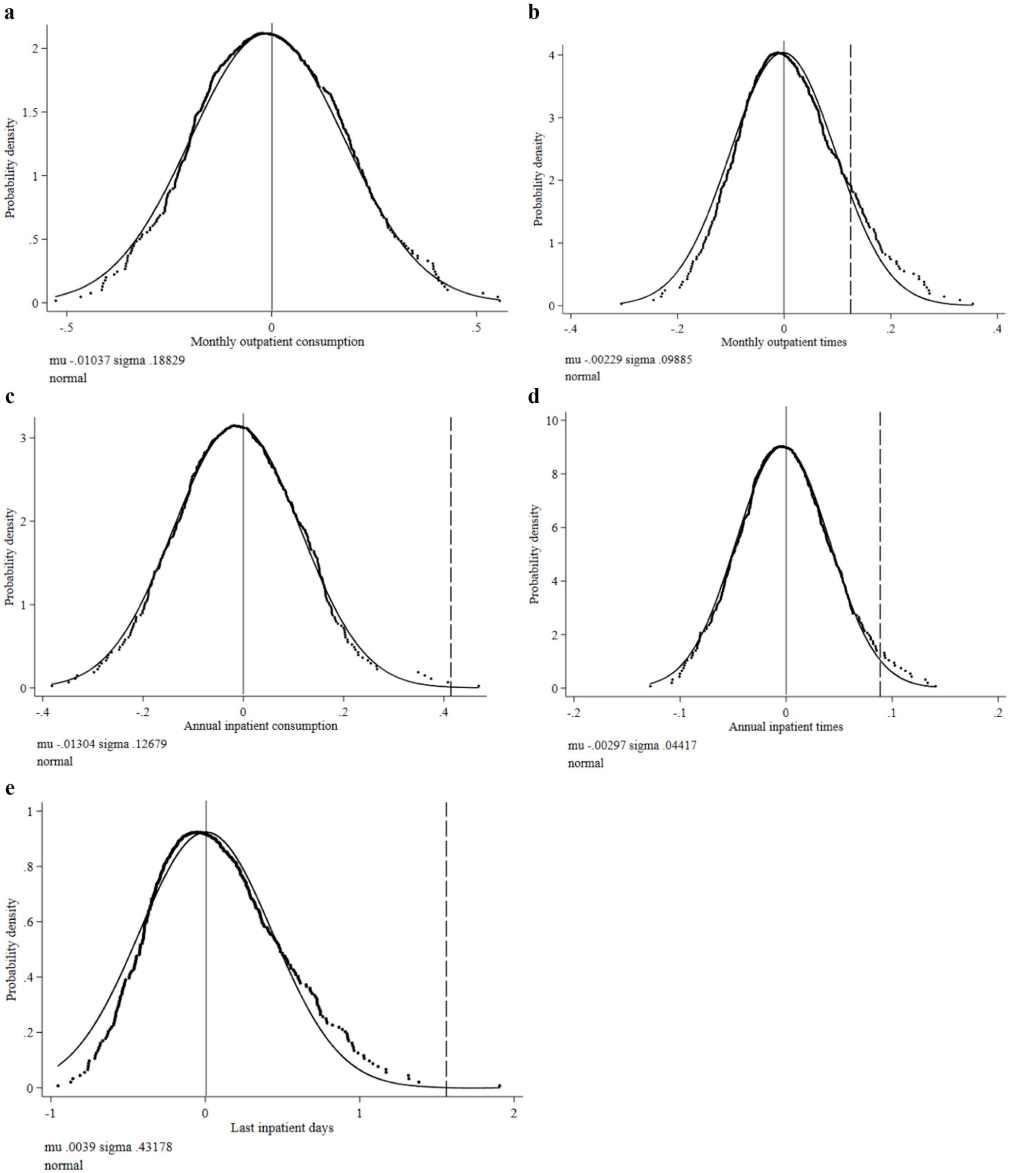
Density distribution of regression coefficient of medical consumption. **(a)** Distribution of monthly outpatient consumption coefficient. **(b)** Distribution of monthly outpatient times coefficient. **(c)** Distribution of annual inpatient consumption coefficient. **(d)** Distribution of annual inpatient times coefficient. **(e)** Distribution of last inpatient days coefficient.

First, we addressed the contingency of the estimation results of the benchmark model. We randomly selected 500 samples from the total for the replacement test, and the coefficient distribution results of these samples are shown in Figure 2. From the distribution results of the five random sampling coefficients in Figures 2a–e, the coefficient distribution shows some similarity to a normal distribution. The dotted vertical lines under Figures 2b–e represent the coefficients of the benchmark test results, which are all located within the coefficient distribution of the random sample test, indicating that the benchmark test results are reliable. The coefficient of the benchmark model is 0.7064 under the monthly outpatient consumption coefficient distribution of random sampling in Figure 2a, which is on the right side of the graph’s coefficient distribution, indicating that the benchmark test results are also robust.

Second, we screened the control group for robustness to ensure the robustness of the benchmark test results and to avoid estimation errors caused by other medical insurance policies implemented during the same period, as well as errors arising from the high proportion of the control group. Additionally, we aimed to confirm that the changes in medical consumption were driven by the LTCI policy. To this end, we conducted a series of robustness tests. We selected the pilot cities of serious illness insurance, the pilot provinces of the integration of the basic medical insurance system for urban and rural residents, and the physical disability of older adults in the pilot city as references to investigate whether the conclusions of this study remain valid after controlling for other medical insurance policy reforms.

1. Considering that the policy for the first batch of 15 national LTCI pilot cities was implemented together in 2016, we selected the 14 pilot cities after 2016 to test robustness. The results are shown in Panel A of Table 6. The treatment still has a significant impact on monthly outpatient consumption, annual hospitalization consumption, annual hospitalization times, and last hospitalization days for older adults, indicating that the benchmark test results are relatively robust.
2. We used cities that implemented serious illness insurance during the same period as the control group. Given that the data used in this study is primarily from 2013 to 2018 and considering the implementation timeline of serious illness insurance in China, we selected the first batch of control groups. Starting from the LTCI pilot in Qingdao in 2012, we selected cities that had introduced serious illness insurance concurrently. By reviewing the pilot documents of serious illness insurance in various cities and adhering to the CHARLS survey samples, we identified the pilot cities for serious illness insurance after 2012 as Beijing, Tianjin, Shijiazhuang, Hohhot, Chifeng, Harbin, Shanghai, Suqian, Lianyungang, and Ningbo.^3^ By controlling for these cities and using a testing method consistent with the benchmark model, the results obtained are shown in Panel B of Table 6. These test results remain robust.
3. Provinces that integrated the basic medical insurance system for urban and rural residents during the same period are considered the control group. Based on the pilot process and completion status of the basic medical insurance integration for urban and rural residents nationwide, the year 2016 is selected as the starting point. The provinces that completed the unification of the basic medical insurance system for urban and rural residents by the end of 2016 are thus selected. Referring to the policy documents and combining the sample characteristics of the CHARLS database, the cities that completed insurance system integration in 2016 mainly include Beijing, Tianjin, Shanghai, Zhejiang, Shandong, Guangdong, Chongqing, and Qinghai, among others.^4^ After the group control and testing using the benchmark model method, the results in Panel C of Table 6 show that the test results are still robust.
4. Based on disabled older adults as the primary focus of this study, and considering the overlap between severe physical disability and severe disability (ADL), we selected severe physical disability as the study group for sample screening. The results are shown in Panel D of Table 6 and remain reliable.

**Table 6:** Test results after screening control group.

Variable	Monthly outpatient consumption (1)	Monthly outpatient times (2)	Annual inpatient consumption (3)	Annual inpatient times (4)	Last inpatient days (5)
Panel A: Cities that implemented LTCI after 2016 were selected as the control group					
Treat	0.6883*** (0.2224)	0.1562 (0.1061)	0.4088*** (0.1401)	0.0935* (0.0533)	1.7039*** (0.4861)
Observations	23,594	23,594	23,594	23,594	23,594
Panel B: Select the cities with serious illness insurance as the control group					
Treat	0.9729*** (0.3222)	−0.1795 (0.1500)	0.5281*** (0.2000)	0.1465* (0.0765)	1.7040** (0.7501)
Observations	9,492	9,492	9,492	9,492	9,492
Panel C: Select the provinces that implement the integration of basic medical insurance system for urban and rural residents at the same time as the control group					
Treat	1.3736*** (0.5121)	−0.2170 (0.2443)	0.6091** (0.2784)	0.0677 (0.1322)	1.9589* (1.1393)
Observations	5,071	5,071	5,071	5,071	5,071
Panel D: Select the group with physical disability as the control group					
Treat	0.7457** (0.3316)	0.1991 (0.1579)	0.2866 (0.2233)	−0.0199 (0.0842)	1.6911* (0.8740)
Observations	7,387	7,387	7,387	7,387	7,387

Robust standard error are given within brackets, ^***^ *p* < 0.01, ^**^ *p* < 0.05, ^*^p < 0.1. Control variable results are not listed.

#### 5.1.2 Medical consumption concentration index and Theil Index

With the year as the control variable, we simultaneously investigated the differences in group medical consumption under the LTCI policy pilot and the results are shown in the upper part of Table 7. From the concentration index (CI) in Table 7, in the control group, monthly outpatient consumption, annual hospitalization consumption, annual hospitalization times, and last hospitalization days are all characterized as “pro-rich,” while monthly outpatient times are characterized as “pro-poor,” which is more conducive to promoting benefits for low-income groups. In the treatment group, the pilot policy of LTCI has significantly altered the fairness of group benefits. Except for the substantial “pro-rich” change in monthly outpatient times, the CI of other medical consumption has decreased, promoting greater fairness in group benefits. The results of the coefficient difference significance test showed that, compared to the control group, the treatment group exhibited more significant changes in monthly outpatient consumption, annual hospitalization consumption, and annual hospitalization times, with the annual hospitalization consumption showing the largest change of −0.1407.

**Table 7:** Statistical results of medical consumption concentration index and Theil Index.

Measure index		Control group	Treatment group	Difference test
CI	Monthly outpatient consumption	0.0989*** (0.0129)	0.0117 (0.0563)	−0.0872 (0.0578)
	Monthly outpatient times	−0.0804*** (0.0081)	0.0228 (0.0321)	0.1032** (0.0331)
	Annual inpatient consumption	0.1669*** (0.0235)	0.0261 (0.0527)	−0.1407** (0.0577)
	Annual inpatient times	0.0703*** (0.0103)	0.0060 (0.0152)	−0.0643*** (0.018)
	Last hospitalization days	0.0537*** (0.0103)	0.0137 (0.0292)	−0.0400 (0.0309)

Measure index		Year of 2013	Year of 2015	Year of 2018
TI	Monthly outpatient consumption (control)	0.0358	0.0300	0.0220
	Monthly outpatient consumption (treat)	0.0000	0.0028	0.0187
	Monthly outpatient times (control)	0.4176	0.3220	0.3743
	Monthly outpatient times (treat)	0.0000	0.0000	0.6577
	Annual inpatient consumption (control)	0.0113	0.0104	0.0084
	Annual inpatient consumption (treat)	0.0000	0.0000	0.0043
	Annual inpatient times (control)	0.1975	0.1684	0.2441
	Annual inpatient times (treat)	0.0000	0.0566	0.3158
	Last hospitalization days (control)	0.2862	0.3073	0.3938
	Last hospitalization days (treat)	0.0000	0.0000	0.3314

*p*-values are given within brackets. CI, concentration index; TI, Theil Index. 0 indicates there is no pilot sample.

From the perspective of the Thiel index regarding whether the LTCI is piloted or not, compared with the trend of the Thiel Index (TI) decreasing year by year in the control group, the TI index in the treatment group increased from 0.0028 in 2015 to 0.0187 in 2018, but it is still smaller than the TI in the control group. That is, after the pilot of the LTCI policy, the inequality of monthly outpatient consumption among residents in the city has decreased, but as the pilot progressed, group inequality has gradually increased. The same pattern is also observed in terms of annual hospitalization consumption and last hospitalization days. However, the monthly outpatient times and annual hospitalization times showed a different trend; the TI of the treatment group was higher than that of the control group, indicating that group inequality was also greater. Thus, the LTCI pilot project has resulted in significant inequality in the number of outpatient times and last hospitalization days.

### 5.2 Disability-related health effects of LTCI policy pilots

#### 5.2.1 Disability-related health effects

##### 5.2.1.1 Benchmark model for health effects

It can be seen from Table 8 that the LTCI policy pilot has significantly affected the health level of older adults. Among them, the impact coefficients on self-assessment health, ADL disability, and the number of serious diseases among older adults were 0.8677, 1.0854, and 0.6668, respectively. This indicates that compared with the control group, self-assessment health, ADL disability, and the number of serious diseases among older adults in the treatment group would increase by 0.8677, 1.0854, and 0.6668 units, respectively. In other words, the indicators reflecting the health level of older adults have significantly worsened; that is, self-assessment health, ADL disability, and the number of serious diseases among older adults in the control sample were significantly worse, increasing by 86.77, 108.54, and 66.68%, respectively. Overall, LTCI has negatively impacted the health of older adults. At the same time, we also conducted a fixed-effects test on cities. The results are shown in Models (4) to (6) in Table 8. The results indicate that the effect of LTCI policies, after accounting for city fixed effects, on disability-related health among older adults remains stable. This is different from existing research conclusions. For example, Ham et al. (34), Han et al. (35), Kashiwagi et al. (36), and An et al. (37) all proposed that LTCI improves the health of beneficiaries. The reasons for the differences are as follows: First, the health effects selected in this study are disability-related health effects, such as ADL disabilities and the number of serious diseases directly associated with disabled individuals in the implementation of China’s LTCI policy, as well as self-assessment health reflecting their overall health level. Since self-assessment health, ADL disability, and the number of serious diseases are directly related to the identification of disabled individuals, at the early stage of policy implementation, the health quality of disabled individuals may decline with the promotion of the LTCI system due to imperfect system design, which can lead to greater moral hazard. Second, the implementation of the LTCI policy induced excessive demand for long-term care services, which led to an increase in the number of individuals actually applying for disability assessments, resulting in a decline in overall health quality, especially the health level directly related to disability assessments.

**Table 8:** Health evaluation benchmark model test results.

Variable	Disability-related health evaluation					
	Benchmark test			City fixed effect		
	Self-assessment health (1)	ADL disability (2)	Serious diseases (3)	Self-assessment health (1)	ADL disability (2)	Serious diseases (3)
Treat	0.8677*** (0.1376)	1.0854*** (0.1301)	0.6668*** (0.0474)	0.9338*** (0.1386)	1.1404*** (0.1312)	0.6602*** (0.0479)
City fixed	NO	NO	NO	Control	Control	Control
Control variable	Control	Control	Control	Control	Control	Control
LR test	428.14^***^	1365.06^***^	-	376.58^***^	1232.74^***^	-
LL/R^2^	−34266.514	−27148.72	0.1368	−34098.248	−26999.2	0.1137
Observations	23,690	23,690	23,690	23,690	23,690	23,690

Robust standard errors are given within brackets, ***p < 0.01, and control variable results are not listed. LL, log likelihood.

##### 5.2.1.2 Robustness test

To ensure that the disability-related health effect results are robust and consistent with the medical consumption effect, we conduct a replacement test and screening control group for the robustness test. First, the results of the displacement test showed that 500 groups were randomly selected for the analysis.

**Figure 3:**
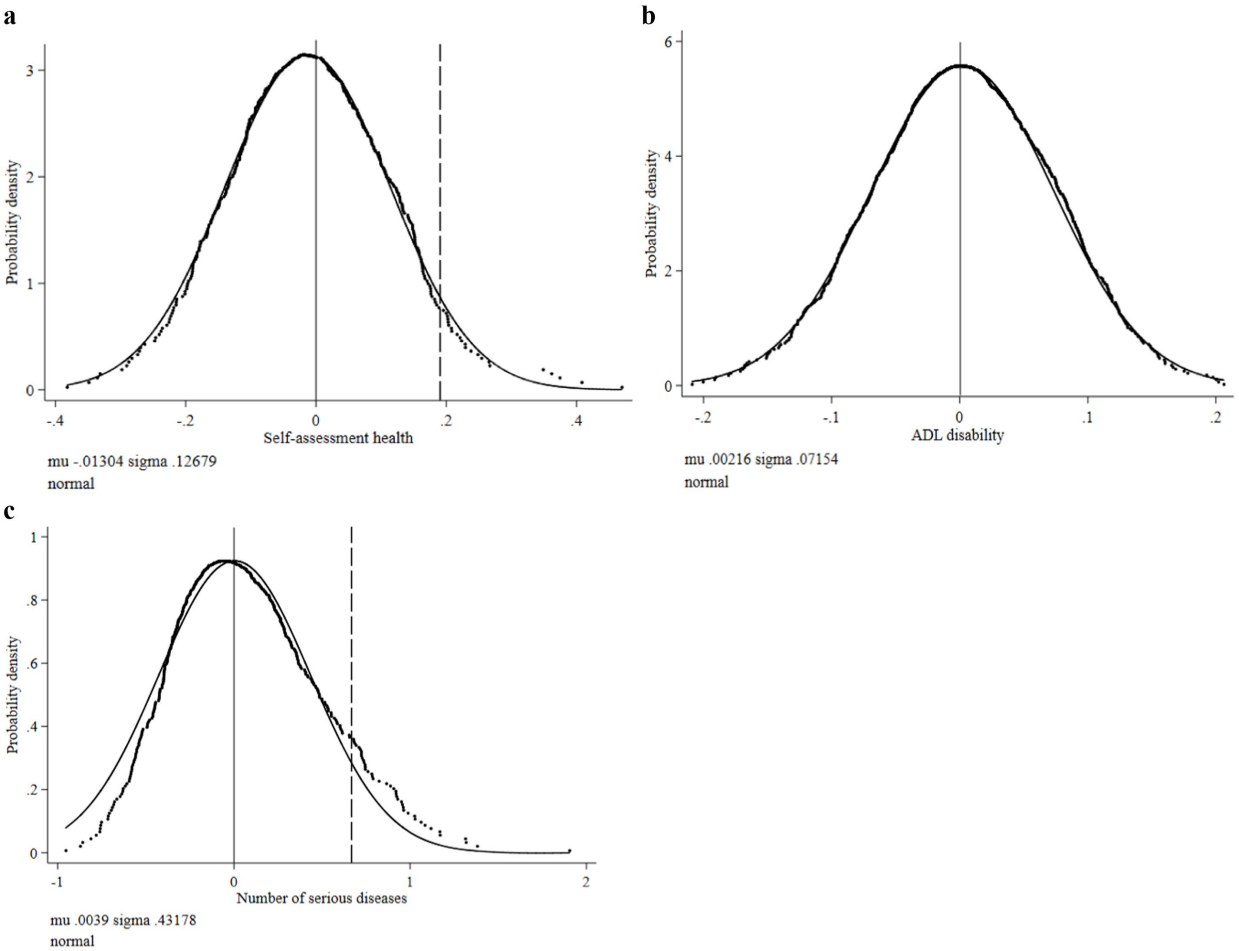
Density distribution of regression coefficients of disability-related health indicators. **(a)** Distribution of self-assessed health coefficient. **(b)** Distribution of ADL disability coefficient. **(c)** Distribution of number of serious diseases coefficient.

The results of Figures 3a–c show that the trend of coefficient distribution and normal distribution under random sampling is highly moderate, and the benchmark test coefficients are all distributed within the random sample distribution, indicating that the benchmark test results are robust. It should be noted that since the ordered model cannot be used for replacement analysis, we convert self-assessed health and ADL disability into a panel effect model for testing. The coefficients of the two under the panel effect are 0.1904 and 0.4647, respectively, as indicated by the dotted line in Figure 3.

Second, consistent with the robustness test of the medical consumption effect, we screen the control group to eliminate the estimation errors caused by the starting time of the LTCI pilot, serious disease insurance pilot, integration of basic medical insurance for urban and rural residents, and the coincidence of physical disability and ADL disability. The method for controlling the screening group is the same as described above, and the results are shown in Table 9. It can be seen from Table 9 that after controlling for the impact of relevant policies, the test results remain robust. Except for some differences between the coefficients and the benchmark model, they are almost consistent with the benchmark model in significance.

**Table 9:** Test results after screening control group.

Variable	Self-assessment health (1)	ADL disability (2)	Number of serious diseases (3)
Panel A: Cities that implemented LTCI after 2016 were selected as the control group			
Treat	0.8329*** (0.1415)	1.0621*** (0.1331)	0.6836*** (0.0488)
Observations	23,594	23,594	23,594
Panel B: Select the cities with serious illness insurance as the control group			
Treat	0.8792*** (0.2038)	1.1888*** (0.1940)	0.6603*** (0.0683)
Observations	9,492	9,492	9,492
Panel C: Select the provinces that implement the integration of basic medical insurance system for urban and rural residents at the same time as the control group			
Treat	1.2208*** (0.3440)	1.1740*** (0.3146)	0.5486*** (0.1030)
Observations	5,071	5,071	5,071
Panel D: Select the group with physical disability as the control group			
Treat	0.7555*** (0.1952)	1.0598*** (0.1947)	0.7123*** (0.0759)
Observations	7,387	7,387	7,387

Robust standard errors are given within brackets, ^***^p < 0.01. Control variable results are not listed.

#### 5.2.2 Disability-related health concentration index and Theil Index

The statistical results of the concentration index and Theil Index of health level are shown in Table 10. In terms of the concentration index, compared with the disability-related health concentration index in the control group, the self-rated health of high-income individuals in the treatment group was more affected, and the number of serious diseases was significantly higher than that in the control group. This indicates that the long-term care insurance policy pilot had a more pronounced effect on optimizing the health of high-income individuals, especially particularly concerning self-rated health and the number of serious diseases. However, as time passed, the disability-related health differences among populations began to decrease. The policy pilot was more conducive to improving the ADL disability of low-income individuals, with a concentration index in the treatment group of −0.0034, indicating that it is more “pro-poor.” However, compared with the control group, this difference was not significant.

**Table 10:** Statistical results of disability-related health concentration index and Theil Index.

Measure index		Control group	Treatment group	Difference test
CI	Self-assessment health	−0.0050*** (0.0007)	0.0011 (0.0022)	0.0061*** (0.0023)
	ADL disability	−0.0009*** (0.0003)	−0.0034* (0.0020)	−0.0025 (0.0020)
	Serious diseases	0.6049*** (0.0058)	0.0301*** (0.0101)	−0.5748*** (0.0117)

Measure index		Year of 2013	Year of 2015	Year of 2018
TI	Self-assessment health (control)	0.0265	0.0299	0.0537
	Self-assessment health (treat)	0.0111	0.0108	0.0305
	ADL disability (control)	0.0093	0.0102	0.0142
	ADL disability (treat)	0.0000	0.0059	0.0340
	Serious diseases (control)	0.0000	0.1834	0.1575
	Serious diseases (treat)	0.0000	0.0000	0.1611

*p*-values are given within brackets. CI, concentration index; TI, Theil Index. 0 indicates that there is no pilot sample.

In terms of the Theil Index, the self-assessment health and ADL disability Theil Index of older adults in the control group gradually increased over time, while the self-assessment health and ADL disability Theil Index of older adults in the treatment group under the same circumstances showed the same characteristics. At the same time, the Theil Index of older adults in the treatment group was lower than that in the control group. However, in 2018, the Theil Index for the number of ADL disabilities and serious diseases among older adults in the treatment group was higher than that of the control group, indicating that the pilot of long-term care insurance has, to some extent, exacerbated the inequality of group health benefits.

## 6 Discussion

The research on the effect of LTCI policy is an important step to further improve the policies of the LTCI system. Focusing on the first batch of LTCI pilot cities in China in 2016 and based on the CHARLS database, this study examines medical consumption, disability-related health, the Concentration index, and the Theil Index as measurement indicators, attempting to empirically investigate the institutional effects of LTCI policy pilots and population coverage. It is revealed that with the expansion of insurance coverage under the LTCI pilot category, the group heterogeneity of medical consumption and disability-related health among older adults is narrowing. Building on the research of Ma et al. (1) and Liu and Hu (2), this study further investigates the institutional effects of differences in China’s LTCI policy pilots differences. Compared with the conclusions of Ma et al. (1) and Liu and Hu (2), this study further refines the accuracy of the policy differences in research methods to ensure that the real influence groups of policies can be included in the basic analysis. The research findings are extensive as well, showing not only whether the LTCI pilot has an impact on residents’ medical consumption but also proving that if the coverage of the pilot policy is wider, the group differences in regional residents’ medical consumption and health levels are smaller. Thus, different types of policy pilots indeed have a significant impact on medical consumption and disability-related health (38). In terms of institutional effect, the research conclusion shows that, from an economic perspective, the policy pilot has significantly and positively affected the overall medical consumption behavior of older adults, with a positive effect being the main outcome (39–41). As a primary supplementary system of medical insurance, the core goal of LTCI is to enhance daily life care and basic medical care services for the disabled older adults (42, 43). In the system design of the various pilot areas, older adult individuals who have been continuously treated for no less than 6 months can enjoy LTCI treatment only after meeting the standards for severe disability. At the same time, to avoid repeated treatment, most pilot cities have set strict restrictions on hospitalization care expenses for disabled individuals. Additionally, the compensation ratio for LTC hospitalization expenses is relatively low compared to the reimbursement proportion of basic medical insurance. Therefore, to obtain corresponding LTCI benefits, disabled individuals and their families may exhibit a tendency toward adverse selection, such as obtaining disability assessment qualifications through long-term hospitalization or outpatient treatment, which results in a positive effect of LTCI on medical consumption. Second, in terms of health effects, the LTCI policy has also significantly reduced disability-related health among disabled individuals, and their self-assessment health, ADL disability, and the number of serious diseases have deteriorated significantly. This diverges from existing research conclusions. For example, studies by relevant scholars based on Japan, South Korea, and China have confirmed that the implementation of LTCI policy has significantly promoted improvements in the health levels of disabled individuals (34–37), which contrasts with the findings of this study. This paper focuses on the impact of the LTCI system at the initial stage of the pilot project. The reason for the differing results from existing studies is that the LTCI policies in various regions of China are not unified at the initial stage of the pilot project, and due to limitations in system design, they do not produce an obvious health improvement effect or a positive promotion effect, but rather a negative reduction effect. On the one hand, the basis for assessing eligibility for health benefits is disability, determined by the overall conditions of individual ability for daily living (ADL) and corresponding diseases. Therefore, as the coverage of the policy pilot gradually expands, more disabled individuals seek LTCI treatment, which increases the likelihood of low-level health evaluations and raises the disease diagnosis rate. Thus, this indicates that self-assessment health levels and the number of serious diseases deteriorate with the expansion of coverage (41, 42, 44). On the other hand, due to the expansion of LTCI coverage, the number of covered beneficiaries is also gradually increasing. More disabled older adult individuals can access LTC services after passing the assessment. However, as the policy primarily targets severely disabled older adults, it cannot effectively promote improvements in the ADL disability of this group and may carry the risk of further deterioration because of the long-term ADL disability. In addition, although the current national LTCI pilot cities of China primarily serve severely disabled older adults, the LTCI still has a certain positive impact, such as reducing the pain perception of severe disabled individuals before dying (38, 39). The above discussion is analyzed from the effectiveness of the pilot system.

Compared to existing studies that focus heavily on the cost control effects of LTCI policy practice (1, 8, 14), this study investigated the impact of the LTCI policy pilot on older adults through a precise policy DID setting, emphasizing the direct medical consumption effects and disability-related health effects of the LTCI policy pilot. The research concluded that high coverage LTCI policy practice in the initial stage of the pilot was not conducive to controlling regional medical expenses or improving disability-related health levels; instead, it resulted in increased short-term medical expenses and worsened disability-related health levels in the group. This result also indicates that the LTCI policy has a medical consumption release effect (5, 15, 28). At the same time, based on the research by Liu and Hu (2), we examined the equity of group benefits influenced by the LTCI policy pilots. The inequality measurement results show that the LTCI policy pilot can promote fairness in group medical consumption to some extent, primarily reflected in annual hospitalization consumption and annual hospitalization times. In addition, LTCI can benefit poorer or low-income individuals to some degree. For example, the CI of ADL disability in the treatment group is significantly higher than that in the control group, and the value is negative. However, as time progresses, its impact on the inequality of ADL disabilities and the number of serious diseases among older adults is increasing, indicating a trend toward exacerbating inequality among groups. This result further enriches the relevant research findings on the effects of the LTCI policy pilot. It also demonstrates that LTCI policy practice not only affects the medical consumption behavior and disability-related health of older adults but also plays an important regulatory role in the equity of regional medical consumption and disability-related health. This finding will provide important empirical support for promoting LTCI policy practice in China and potentially in other countries around the world.

Based on the existing research, this study makes a breakthrough in investigating the impact of LTCI policy pilots on medical consumption and disability-related health levels. We select 3 years of follow-up survey data from CHARLS for empirical analysis. Its important contribution lies in the investigation of policy pilot differences. The research conclusion enriches the understanding of the effects of different LTCI policy pilots and their impacts on the fairness of group benefits. Through the findings of this study, the following policy implications can be listed: First, under the increasing pressure of medical expenses worldwide, implementing an LTCI policy focused on older adults will help alleviate this financial burden. However, this requires fully leveraging the LTC guarantee function to effectively save medical resources, improve their utilization rate, and enhance residents’ disability-related health levels. Second, in the implementation process of the LTCI policy pilot, designing a policy with wide coverage will help improve the utilization rate of medical resources for disabled older adults and reduce the repetitive use of these resources. However, in practice, there is still a phenomenon of rising medical expenses and deteriorating disability-related health levels in the short term. Therefore, it is necessary to optimize the LTCI pilot policy and gradually include beneficiaries from other groups, not just those who are severely disabled, while also avoiding or reducing the moral hazard caused by repeated medical and nursing compensation. Third, in the process of policy promotion, the government should consider expanding coverage to moderately disabled individuals based on the actual operation of the system. In the future, to protect the rights and interests of disabled persons and ensure the fairness of LTCI system benefits, the government should learn from the design of LTCI systems in Japan and other countries, including low disability-level individuals in disability prevention guarantees and providing them with necessary preventive measures, thereby aiming to reduce the incidence of disability through policy. Finally, in implementing the LTCI policy, we should not only focus on the daily life security of residents with ADL disabilities but also consider the role of LTCI in controlling medical expenses in a country or region, as well as its contribution to improving residents’ disability-related health levels. Thus, the implementation of the LTCI policy can address the unfairness of group benefits caused by medical insurance policies.

Besides, there are some limitations in the research design of this study. (1) We use the panel model to construct policy variables. We explored both the coverage variation of policy implementation and the changes before and after the implementation time. Thus, changes may occur in the same area before and after the policy is implemented. It should be noted that we first chose individuals over 60 as the main subjects, as this group has a higher probability of medical consumption than others. After controlling for individual characteristics and the impact of regional medical insurance policies, it is feasible to investigate the effects of the pilot LTCI policy on medical consumption. However, this process cannot completely eliminate bias in sample selection. (2) Another limitation of this study is that we can only use the tracking survey of CHARLS to match the data and obtain information on the actual beneficiaries of China’s current LTCI policy pilot, which may introduce certain errors in the research findings. Therefore, in further research, additional robustness tests can be conducted based on the data obtained from the LTCI pilot area to ensure the reliability of this research conclusion.

## 7 Conclusion

The main conclusions of this study are as follows: (1) The LTCI policy pilot significantly impacts the short-term medical consumption behavior of severely disabled older adults and improves the overall medical consumption behavior of the disabled. The effect of this policy pilot is consistent across different cities, indicating that the LTCI policy has a medical consumption release effect. (2) The LTCI policy pilot will lead to a comprehensive deterioration of disability-related health among disabled older adults in the short term. For example, key indicators of disability assessment, such as ADL disability degree and the number of serious diseases, will worsen due to the implementation of short-term policies, that is, short-term LTCI policies also increase disability risk. (3) The fairness of group medical consumption and disability-related health benefits under the LTCI policy pilot is limited. In the short term, it may reduce differences in medical consumption or disability-related health among groups, but in the long term, its impact on the “rich” remains greater than on the “poor,” and the disparities between groups will likely increase over time with the policy pilot.

## Data Availability

Publicly available datasets were analyzed in this study. This data can be found at: http://charls.pku.edu.cn/.

## Footnotes

^1^ http://www.gov.cn/xinwen/2016-07/08/content_5089283.htm

^2^ http://www.gov.cn/zhengce/zhengceku/2020-11/05/content_5557630.htm

^3^Combined with the CHARLS survey sample, it is determined that the cities of pilot serious illness insurance after 2012 mainly include Beijing, Tianjin, Shijiazhuang, Huhehaote, Chifeng, Haerbin, Shanghai, Suqian, Lianyungang, Ningbo, Hangzhou, Lu′an, Zhangzhou, Fuzhou, Ningde, Jingdezhen, Nanchang, Binzhou, Qingdao, Jinan, Zaozhuang, Linyi, Liaocheng, Weihai, Weifang, Dezhou, Anyang, Luoyang, Zhengzhou, Jingmen, Xiangyang, Changde, Foshan, Guangzhou, Chongqing, Chengdu, Nanchong, Yibin, Ganzi, Kunming, Baoji, Hanzhong, Dingxi, and Haidong.

^4^According to the CHARLS survey samples, Suzhou, Xuzhou, Taizhou, Yancheng, Suqian, Yangzhou, and Lianyungang in Jiangsu Province were selected for control.

## References

[1] Ma C Yu QW Song Z. Long-term care insurance, the control of medical expenses and "value-based health care". China Industrial Econ. (2019) 12:42–59. doi: 10.19581/j.cnki.ciejournal.2019.12.003

[2] Liu H Hu T. Evaluating the long-term care insurance policy from medical expenses and health security equity perspective: evidence from China. Arch Public Health. (2022) 80:3. doi: 10.1186/s13690-021-00761-7, PMID: 34983634 PMC8725500

[3] Lin HR Imanaka Y. Effects of copayment in LTCI on long-term care and medical care expenditure. J Am Med Dir Assoc. (2020) 21:640–646.e5. doi: 10.1016/j.jamda.2019.08.021, PMID: 31623988

[4] Liu H. Formal and informal care: complementary or substitutes in care for elderly people? Empirical evidence from China. SAGE Open. (2021) 11:1–16. doi: 10.1177/21582440211016413, PMID: 40297624

[5] Boo S Lee J Oh H. Cost of care and pattern of medical care use in the last year of life among long-term care insurance beneficiaries in South Korea: using national claims data. Int J Environ Res Public Health. (2020) 17:9078. doi: 10.3390/IJERPH17239078, PMID: 33291790 PMC7730132

[6] Feng J Wang Z Yu Y. Does long-term care insurance reduce hospital utilization and medical expenditures? Evidence from China. Soc Sci Med. (2020) 258:113081. doi: 10.1016/j.socscimed.2020.113081, PMID: 32540515

[7] Kim HB Lim W. Long-term care insurance, informal care, and medical expenditures. Mathematica Policy Res Rep. (2015) 125:128–42. doi: 10.1016/j.jpubeco.2014.12.004, PMID: 40343189

[8] Chang S Yang W Deguchi H. Care providers, access to care, and the long-term care nursing insurance in China: an agent-based simulation. Soc Sci Med. (2020) 244:112667. doi: 10.1016/j.socscimed.2019.112667, PMID: 31734601

[9] Choi JW Park EC Lee SG Park S Ryu HG Kim TH. Does long-term care insurance reduce the burden of medical costs? A retrospective elderly cohort study: does LTCI lower burden of medical cost? Geriatr Gerontol Int. (2018) 18:1641–6. doi: 10.1111/ggi.13536, PMID: 30311345

[10] Schut FT Van Den Berg B. Sustainability of comprehensive universal long-term care insurance in the Netherlands. Soc Policy Adm. (2010) 44:411–35. doi: 10.1111/j.1467-9515.2010.00721.x

[11] Swift R. The relationship between health and GDP in OECD countries in the very long run. Health Econ. (2011) 20:306–22. doi: 10.1002/hec.1590, PMID: 20217835

[12] Theobald H Hampel S. Radical institutional change and incremental transformation: Long-term care insurance in Germany. New York: Springer (2014).

[13] Shimizutani S. The future of long-term Care in Japan. Asia Pacific Rev. (2014) 21:88–119. doi: 10.1080/13439006.2014.925199

[14] Liu H. Research on disability grading based on ICF functional framework: empirical evidence from Zhejiang province, China. Front Public Health. (2021) 9:616180. doi: 10.3389/fpubh.2021.616180, PMID: 34046386 PMC8144326

[15] Bascans JM Courbage C Dafny L Oros C. Means-tested public support and the interaction between long-term care insurance and informal care. Int J Health Econ Manag. (2017) 17:113–33. doi: 10.1007/s10754-016-9206-4, PMID: 28013398

[16] Narayan PK Narayan S Smyth R. Is health care really a luxury in OECD countries? Evidence from alternative price deflators. Appl Econ. (2011) 43:3631–43. doi: 10.1080/00036841003670788

[17] Roquebert Q Tenand M. Pay less, consume more? The price elasticity of home care for the disabled elderly in France. Health Econ. (2017) 26:1162–74. doi: 10.1002/hec.3531, PMID: 28660657

[18] Rothgang H. Social insurance for long-term care: an evaluation of the German model. Soc Policy Adm. (2010) 44:436–60. doi: 10.1111/j.1467-9515.2010.00722.x

[19] Rapp T Grand A Cantet C Andrieu S Coley N Portet F. Public financial support receipt and non-medical resource utilization in Alzheimer's disease results from the PLASA study. Soc Sci Med. (2011) 72:1310–6. doi: 10.1016/j.socscimed.2011.02.039, PMID: 21463914

[20] Nadash P Shih YC. Introducing social insurance for long-term care in Taiwan (Chinese): key issues. Int J Soc Welf. (2013) 22:69–79. doi: 10.1111/j.1468-2397.2011.00862.x

[21] Zhang Z Wang J Jin M Li M Zhou L Jing F. Can medical insurance coverage reduce disparities of income in elderly patients requiring long-term care? The case of the People's Republic of China. Clin Interv Aging. (2014) 9:771–7. doi: 10.2147/CIA.S58771, PMID: 24855346 PMC4020881

[22] Yasutake T Takashi W Kemmyo S Zhang S Sugawara Y Tsuji I. Effects of a community-based program for oral health and nutrition on cost-effectiveness by preventing disability in Japanese frail elderly: a quasi-experimental study using propensity score matching. J Am Med Dir Assoc. (2017) 18:678–85. doi: 10.1016/j.jamda.2017.02.014, PMID: 28412165

[23] Ba Ltagi BH Moscone F. Health care expenditure and income in the OECD reconsidered: evidence from panel data. Econ Model. (2010) 27:804–11. doi: 10.1016/j.econmod.2009.12.001

[24] Shenkman E Knapp C Sappington D Vogel B Schatz D. Persistence of high health care expenditures among children in Medicaid. Med Care Res Rev. (2007) 64:304–30. doi: 10.1177/1077558707299864, PMID: 17507460

[25] Newhouse JP. Medical care costs: how much welfare loss? J Econ Perspect. (1992) 6:3–21. doi: 10.1257/jep.6.3.3, PMID: 10128078

[26] Jung WS Yim ES. The effect on health care utilization of the non-use of beneficiaries of long-term Care Insurance Service-around of Geriatric Hospital's medical. Cost J Korea Acad. (2015) 16:7463. doi: 10.5762/KAIS.2015.16.11.7463

[27] Nemoto M Yabushita N Kim MJ Matsuo T Seino S Tanaka K. Assessment of vulnerable older adults' physical function according to the Japanese long-term care insurance (LTCI) system and fried's criteria for frailty syndrome. Arch Gerontol Geriatr. (2012) 55:385–91. doi: 10.1016/j.archger.2011.10.004, PMID: 22100110

[28] Okma K Gusmano MK. Aging, pensions and long-term care: what, why, who, how? Comment on "financing long-term care: lessons from Japan" commentary. Int J Health Policy Manag. (2020) 9:218–21. doi: 10.15171/IJHPM.2019.117, PMID: 32563225 PMC7306113

[29] Shen S Li F Tanui JK. Long-term care insurance in China: public or private? Soc Work Health Care. (2014) 53:679–92. doi: 10.1080/00981389.2014.925999, PMID: 25133301

[30] Zhang H Zhang X Zhao Y Huang J Liu W. Impact of formal care use on informal care from children after the launch of long-term care insurance in Shanghai, China. Int J Environ Res Public Health. (2020) 17:29–38. doi: 10.3390/ijerph17082938, PMID: 32344522 PMC7216006

[31] Liu H Hu T. How does air quality affect residents’ life satisfaction? Evidence based on multiperiod follow-up survey data of 122 cities in China. Environ Sci Pollut Res. (2021) 28:61047–60. doi: 10.1007/s11356-021-15022-x 34169414

[32] Liu H Wang M. Socioeconomic status and ADL disability of the older adults: cumulative health effects, social outcomes and impact mechanisms. PLoS One. (2022) 17:e0262808. doi: 10.1371/journal.pone.0262808, PMID: 35143499 PMC8830695

[33] Grossman M. On the concept of health capital and the demand for health. J Polit Econ. (1972) 80:223–55. doi: 10.1086/259880

[34] Ham OK Cho I Kim D Suh M. Factors associated with medical expenses among long-term care insurance recipients aged 65 years or older in Korea. Nurs Health Sci. (2024) 26:e70020. doi: 10.1111/nhs.70020, PMID: 39721721

[35] Han X Wang H Du X. The impact of long-term care insurance on the utilization of inpatient service: evidence and mechanisms in China. Health Econ. (2024) 33:2778–97. doi: 10.1002/hec.4896, PMID: 39267463

[36] Kashiwagi M Kashiwagi K Morioka N Abe K. Last year of life care transitions between long-term care insurance services in Japan: analysis of long-term care insurance claims data. Geriatr Gerontol Int. (2024) 24:883–90. doi: 10.1111/ggi.14944, PMID: 39081082

[37] An R Xiu S Yang X Wang S. The impact of long-term care insurance on the health status and healthcare expenditure of older adults in China. Front Psych. (2025) 15:1514603. doi: 10.3389/fpsyt.2024.1514603, PMID: 39872431 PMC11770830

[38] Kim H Kwon S. A decade of public long-term care insurance in South Korea: policy lessons for aging countries. Health Policy. (2021) 125:22–6. doi: 10.1016/j.healthpol.2020.11.003, PMID: 33189411

[39] Chen L Xu X. Effect evaluation of the long-term care insurance (LTCI) system on the health care of the elderly: a review. J Multidiscip Healthc. (2020) 13:863–75. doi: 10.2147/JMDH.S270454, PMID: 32922026 PMC7457853

[40] Chon Y. The expansion of the Korean welfare state and its results-focusing on long-term care insurance for the elderly. Soc Policy Adm. (2015) 48:704–20. doi: 10.1111/spol.12092, PMID: 40335190

[41] Lei X Bai C Hong J Liu H. Long-term care insurance and the well-being of older adults and their families: evidence from China. Soc Sci Med. (2022) 296:114745. doi: 10.1016/j.socscimed.2022.114745 35093795

[42] Chen H Ning J. The impacts of long-term care insurance on health care utilization and expenditure: evidence from China. Health Policy Plan. (2022) 11:6. doi: 10.2991/jracr.k.210310.001, PMID: 35032390

[43] Soga Y Murata F Maeda M Fukuda H. The effects of raising the long-term care insurance co-payment rate on the utilization of long-term care services. Geriatr Gerontol Int. (2020) 20:685–90. doi: 10.1111/ggi.13935, PMID: 32445437

[44] Sohn M O'Campo P Muntaner C Chung H Choi M. Has the long-term care insurance resolved disparities in mortality for older Koreans? Examination of service type and income level. Soc Sci Med. (2020) 247:112812. doi: 10.1016/j.socscimed.2020.112812, PMID: 32066015

